# Characterization of the complete mitochondrial genome of *Dactylopterus volitans* (Syngnathiformes, Dactylopteridae)

**DOI:** 10.1080/23802359.2019.1704651

**Published:** 2019-12-23

**Authors:** Ha Yeun Song, Young Ji Choi, Seonmi Jo, Bora Kim, Seung-Hyun Jung, Jong Su Yoo, Dae-Sung Lee

**Affiliations:** Department of Genetic Resources Research, National Marine Biodiversity Institute of Korea, Seocheon-gun, Republic of Korea

**Keywords:** Mitochondrial genome, Syngnathiformes, Dactylopteridae, *Dactylopterus volitans*

## Abstract

The complete mitochondrial genome was determined for the flying gurnard *Dactylopterus volitans* belonging to the family Dactylopteridae. The total length of the *D. volitans* mitochondrial genome is 16,632 bp, which consists of 13 protein-coding genes, 22 tRNA genes, two rRNA genes, and a control region. It has the typical vertebrate mitochondrial gene arrangement. Phylogenetic analysis using mitochondrial genomes of 20 species showed that *D. volitans* formed a well-supported monophyletic group with other Dactylopteridae species.

The flying gurnard *Dactylopterus volitans* (Syngnathiformes, Dactylopteridae) notable for their tremendously enlarged and colorful pectoral fins is a bottom-dwelling fish of tropical waters in the Atlantic Ocean (Nelson et al. 2016). It is listed as least concern in the IUCN Red List of Threatened Species (Carpenter et al. 2015). Their phylogenetic position of this group, the family Dactylopteridae was changed from Scorpaeniformes to Syngnathiformes by Nelson (2016) based on the molecular evidence (Betancur et al. 2013; Near et al. 2013; Song et al. 2014); however, their taxonomic position remains controversial. In the current study, we first determined the complete mitochondrial DNA sequence of *D. volitans* and to analyze the phylogenetic relationship of this species with members of Syngnathiformes.

The *D. volitans* specimen used in this study was collected from high seas of the Western Central Atlantic (8.30 N, 14.00 W). Total genomic DNA was extracted from tissue of the specimen, which has been deposited in the National Marine Biodiversity Institute of Korea (Voucher No. MABIK 0001979). The mitogenome was sequenced and assembled using Illumina Hiseq 4000 sequencing platform (Illumina, San Diego, CA) and *SOAP denovo* assembler at Macrogen Inc. (Korea), respectively. The complete mitochondrial genome was annotated using MacClade ver. 4.08 (Maddison and Maddison 2005) and tRNAscan-SE 2.0 (Lowe and Chan 2016).

The complete mitochondrial genome of *D. volitans* (GenBank accession no. LC512458) is 16,632 bp long, and includes 13 protein-coding genes, 22 tRNA genes, 2 rRNA genes, and a control region. The *ND6* gene and eight tRNA genes are encoded on the light strand. The overall base composition of the heavy strand is 27.92% A, 30.20% C, 16.29% G, and 25.59% T. Similar to the mitogenomes of other vertebrates, the AT content is higher than the GC content (Saccone et al. 1999). All tRNA genes can fold into a typical cloverleaf structure, with lengths ranging from 66 to 74 bp. The 12S rRNA (956 bp) and 16S rRNA genes (1,692 bp) are located between tRNA^Phe^ and tRNA^Val^ and between tRNA^Val^ and tRNA^Leu(UUR)^, respectively. Of the 13 protein-coding genes, 12 begin with an ATG start codon; the exception being the *COI* gene, which start with GTG. The stop codon of the protein-coding genes is TAA in *ND1*, *COI*, *ATP8*, *ND4L*, *ND5* and *ND6*; TA in ND2, ATP6 and *COIII* and T in the remaining four genes. The control region (870 bp) is located between tRNA^Pro^ and tRNA^Phe^.

Phylogenetic trees were constructed by the maximum-likelihood method using MEGA 7.0 software (Kumar et al. 2016) for the newly sequenced genome and a further 19 complete mitochondrial genome sequences downloaded from the National Center for Biotechnology Information. We confirmed that Dactylopteridae including *D. volitans* formed a monophyletic group together with other members of Syngnathiformes (Fistulariidae and Centriscidae) and this group formed a sister-group relationship with the family Pegasidae species with high statistical support (Figure 1).

**Figure 1.**
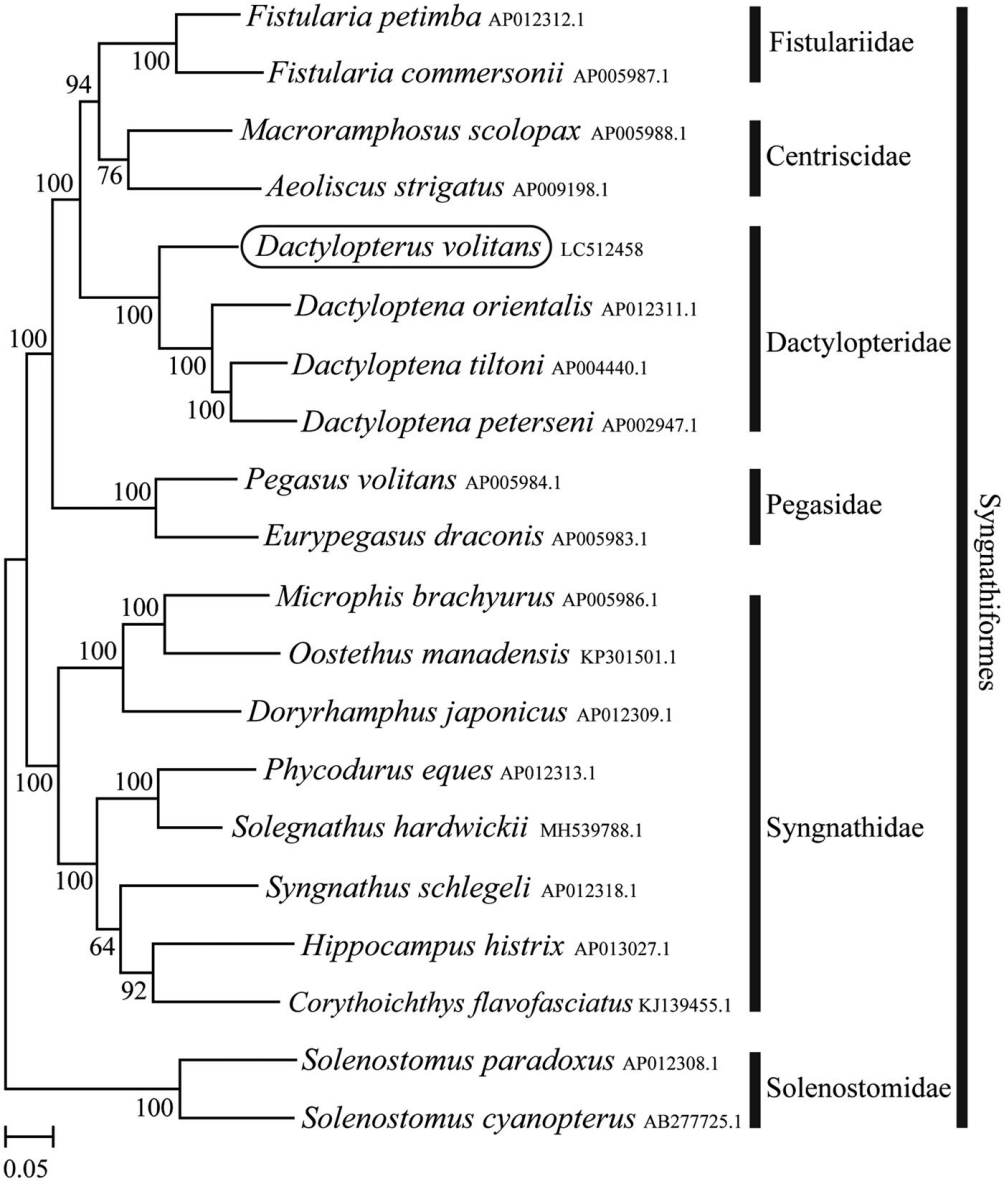
Phylogenetic position of *Dactylopterus volitans* based on a comparison with the complete mitochondrial genome sequences of 19 species. The analysis was performed using MEGA 7.0 software. The accession number for each species is indicated after the scientific name.
